# Measuring moral distress in health professionals using the MMD-HP-SPA scale

**DOI:** 10.1186/s12910-024-01041-z

**Published:** 2024-04-03

**Authors:** Eloy Girela-Lopez, Cristina M. Beltran-Aroca, Jaime Boceta-Osuna, Dolores Aguilera-Lopez, Alejandro Gomez-Carranza, Manuel Lopez-Valero, Manuel Romero-Saldaña

**Affiliations:** 1 Section of Legal and Forensic Medicine. Faculty of Medicine and Nursing, University of Córdoba, Av. Menéndez Pidal s/n, 14004 Córdoba, Spain; 2 Unidad de Cuidados Paliativos, Hospital Universitario Virgen Macarena, Sevilla, Spain; 3 Distrito Sanitario Córdoba-Guadalquivir, Córdoba, Spain; 4 Unidad de Cuidados Intensivos, Hospital Universitario Poniente, Almería, Spain; 5 Dispositivo de Cuidados Críticos y Urgencias, Distrito Sanitario Córdoba-Guadalquivir, Córdoba, Spain; 6 Department of Nursing, Pharmacology and Physiotherapy. Faculty of Medicine and Nursing, University of Cordoba, Córdoba, Spain

**Keywords:** Moral distress, Health professionals, MMD-HP-SPA, COVID-19, Ethical issues

## Abstract

**Background:**

Moral distress (MD) is the psychological damage caused when people are forced to witness or carry out actions which go against their fundamental moral values. The main objective was to evaluate the prevalence and predictive factors associated with MD among health professionals during the pandemic and to determine its causes.

**Methods:**

A regional, observational and cross-sectional study in a sample of 566 professionals from the Public Health Service of Andalusia (68.7% female; 66.9% physicians) who completed the MMD-HP-SPA scale to determine the level of MD (0-432 points). Five dimensions were used: i) Health care; ii) Therapeutic obstinacy-futility, iii) Interpersonal relations of the Healthcare Team, iv) External pressure; v) Covering up of medical malpractice.

**Results:**

The mean level of MD was 127.3 (SD=66.7; 95% CI 121.8-132.8), being higher in female (135 vs. 110.3; *p*<0.01), in nursing professionals (137.8 vs. 122; *p*<0.01) and in the community setting (136.2 vs. 118.3; *p*<0.001), with these variables showing statistical significance in the multiple linear regression model (*p*<0.001; r^2^=0.052). With similar results, the multiple logistic regression model showed being female was a higher risk factor (OR=2.27; 95% CI 1.5-3.4; *p*<0.001). 70% of the sources of MD belonged to the dimension "Health Care" and the cause "Having to attend to more patients than I can safely attend to" obtained the highest average value (Mean=9.8; SD=4.9).

**Conclusions:**

Female, nursing professionals, and those from the community setting presented a higher risk of MD. The healthcare model needs to implement an ethical approach to public health issues to alleviate MD among its professionals.

## Background

Moral distress (MD) is defined as the psychological damage which arises when people are forced to witness or carry out decisions or actions that go against their fundamental moral values [1, 2]. Hamric and other researchers have further added nuances to this definition, stating that MD not only depends on external impediments but is a function of many factors, one of which is moral sensitivity [3]. Moreover, repeated and unaddressed situations of moral distress over time cause a gradual “crescendo” of moral residue that undermines the professional commitment of healthcare providers [4].

The distinctive feature of MD compared with other concepts such as burnout or post-traumatic stress is the perception of a breach of duty and professional integrity, and the feeling that one is prevented from doing what is ethically correct [5]. Nevertheless, these concepts are closely linked, in that MD caused by situations of constant conflict can lead to emotional exhaustion (burnout) and job dissatisfaction in the health professional [6], and there are studies that clearly associate MD with burnout [7]. Its effects on both physical and mental health have been described, with feelings of anger and guilt, symptoms of depression, anxiety, sadness, headaches, digestive and sleep disorders, frustration, a sense of impotence, stress and a negative perception of self-image highlighted, among others [8].

MD is a problem which arises in routine clinical practice as healthcare professionals, particularly physicians and nurses, are exposed to difficulties, lengthy shifts, stress and great responsibility. Their job is to witness and help people overcome life's most serious challenges: death and dying, suffering, loss and pain [9]. These are challenges which are inherent to the profession and often require the professional to take difficult decisions when faced with uncertainty [2].

The incidence of MD is influenced by various factors. Firstly, it is affected by working conditions when there is an unethical climate in the health institution itself, a low level of collaboration between colleagues or a lack of ethical debate [10–14]. Other more personal factors have also been associated, including lack of understanding, lack of knowledge, low levels of assertiveness, the perception of helplessness or a lack of self-confidence [4]. To this it can be added the experience of situations produced by the COVID-19 pandemic, in which scarce resources had to be prioritized, which led to inevitable delays in care for other health issues which could lead to serious harm or the need to make difficult ethical-clinical decisions without suitable advice [15]. Another key factor has also been the distress caused by not being able to provide the necessary emotional support to suffering patients due to preventive measures. It is for all these reasons that the level of MD has risen among health professionals around the world [2, 16, 17].

The focus of attention has been placed on MD as it is considered one of the causes of lower quality in patient care. In fact, it is one of the most commonly researched psycho-physical conditions, with the aim of improving not only the well-being of professionals, but also the sustainability of health institutions and the care provided to patients [18].

Epstein et al. [19] designed a 27-item MD scale (Measure of Moral Distress for Healthcare Professionals, MMD-HP), revising the earlier MDS-R scale developed by Hamric et al. [4] This instrument has proved its worth in measuring MD and has been validated first in the USA and later in Japan [20] and Spain [21, 22].

The aim of the present study was to evaluate the prevalence and identify the predictive factors associated with the MD among health professionals during the pandemic, and to compare them with other existing studies on the subject [23–25], in an attempt to find what causes most MD in our sample, as well as plausible explanations for the causes of MD.

## Methods

A regional, observational, cross-sectional epidemiological study was carried out to determine the level of MD and its predictive or associated variables among health professionals in Andalusia (Spain). The study was conducted between October 2021 and January 2022.

The reference population was made up of 45,440 health workers, physicians and nurses from primary health care and hospital care, belonging to the Andalusian Public Health Service. To calculate the sample size, the following were taken as expected values: a standard deviation of 60 points [20, 26], an absolute precision of 6, a 95% CI and a design effect equal to 1, resulting in a minimum sample of 381 health professionals.

The recruitment of participants was carried out from 7 reference hospitals and 8 primary care health districts in the Andalusia region. The sample was obtained through consecutive sampling by completing an online form.

### Eligibility criteria

- Inclusion criteria: i) being a physician or nurse in the Andalusian Public Health System and belonging to the following professional services: Clinical Management Units (CMUs) of Primary Health Care (PHC), Palliative Care, Intensive Care (ICU), Internal Medicine, Pneumology and Emergency Ward; ii) signing the informed consent prior to completing the questionnaire.
- Exclusion criteria: Having had less than one year’s effective experience in care work or being a first-year resident in any medical or nursing speciality.

### Study variables and measurement instruments

Resultant variable**.** Moral Distress: measured using the MMD-HP SPA scale with an identical structure to the original scale devised by Epstein et al. [19] made up of 27 items categorized by Likert-type responses which record the frequency of occurrence for each question, with scores ranging from 0 (never) to 4 (very frequently), and the level of MD with values ranging from 0 (none) to 4 (maximum distress). For each item, the frequency was multiplied by the level of distress and the global value of the scale was obtained by adding the scores for all the items (0-432 points).

The five dimensions of the scale proposed by Girela-López et al. were used: i) Health care, ii) Therapeutic obstinacy-futility, iii) Interpersonal relations of the Health Care Team, iv) External pressure, v) Covering up medical malpractice [22].

Explanatory variables**.** Age (years), sex, profession (physician, nurse), experience (years), care setting (hospital, community) and CMU (PHC, Palliative Care, ICU, Internal Medicine, Pneumology and Emergency Ward).

### Ethical and legal aspects

This research study obtained the authorization from the corresponding Córdoba Research Ethics Committee (Spain) (Document No.5158, dated 28/09/2021). All the subjects participating in the study agreed to take part in it by completing and registering the informed consent form.

### Statistical analysis

The quantitative variables are represented by their mean, range, and standard deviations, as well as the median and interquartile range (IQR), while the qualitative variables are represented by their absolute and relative frequency.

Student's T test was used to compare the means in independent groups and the Analysis of variance (ANOVA) test for the comparison of 3 or more independent arithmetic means. A double multivariate analysis (multiple linear and multiple logistic regression) was carried out to discover the degree of discrimination, prediction and association of the independent variables on MD. The model’s discriminant capacity was measured by calculating the area under the curve, and the diagnostic accuracy through the validity and security indicators (sensitivity, specificity, predictive values and validity index). Because the MMD-HP-SPA scale lacks a cutoff point, the mean MMD was used to dichotomize the outcome variable in the logistic regression.

To support the calculation and statistical analysis, the SPSS v.22 and EPIDAT v.4.2 statistical programs were used. The level of statistical significance was established at an alpha error of less than 5% for all the statistical contrasts, and the level of security was set at 95% to create the confidence intervals.

## Results

### Description of the study sample

The study sample consisted of 566 health professionals, of whom 389 were females (68.7%) and 382 physicians (66.9%). The sociodemographic variables of the participants are shown in Table 1. For the variables with missing data, the assumption of random distribution of data was checked using the MCAR-test, finding no significant differences.

**Table 1:** Characteristics of the study sample

**Variable**	**Total** ***N*=566**	**Males** ***N*=177**	**Females** ***N*=389**	***P***
**Age *N*=562**				
Mean age (SD) (years)	49 (10.9)	51.2 (10.9)	48 (10.7)	<0.01
Age categories				
<35	80 (14.2%)	20 (25%)	60 (75%)	<0.01
35-50	199 (35.4%)	50 (25.1%)	149 (74.9%)	
>50	283 (50.4%)	106(37.5%)	177 (62.5%)	
**Work Experience *N*=502**				
Mean experience (SD) (years)	17.3 (11)	19.7 (10.9)	16.2 (10.7)	<0.01
Work experience (SD) categories				
≦10	167 (33.3%)	43 (25.7%)	124 (74.3%)	<0.05
11-20	138 (27.5%)	43 (31.2%)	95 (68.8%)	
>20	197 (39.2%)	78 (39.6%)	119 (60.4%)	
**Professional Position *N*=566**				
Physician	377 (66.6%)	143 (37.9%)	234 (62.1%)	<0.001
Nurse	189 (33.4%)	34 (18%)	155 (82%)	
**Professional Service *N*=555**				
Palliative Care	36 (6.5%)	8 (22.2%)	28 (77.8%)	<0.01
Internal Medicine	58 (10.5%)	19 (32.8%)	39 (67.2%)	
Pneumology	50 (9%)	25 (50%)	25 (50%)	
ICU^a^	95 (17.1%)	20 (21.1%)	75 (78.9%)	
Emergency Ward	62 (11.1%)	27 (43.5%)	35 (56.5%)	
PHC^b^	254 (45.8%)	74 (29.1%)	180 (70.9%)	
**Work Setting *N*=524**				
Hospital	239 (45.6%)	72 (30.1%)	167 (69.9%)	0.53
Community	285 (54.4%)	87 (30.5%)	198 (69.5%)	

^a^*ICU* Intensive Care Unit

^b^*PHC* Primary Health Care

Age and experience were higher in males than in females (*p*<0.01). The distribution of females in the nursing workforce was significantly higher than in medicine (*p*<0.001). However, as regards the work setting (hospital vs. community), no differences were found according to sex (*p*=0.53).

### Level of moral distress

**Fig. 1:**
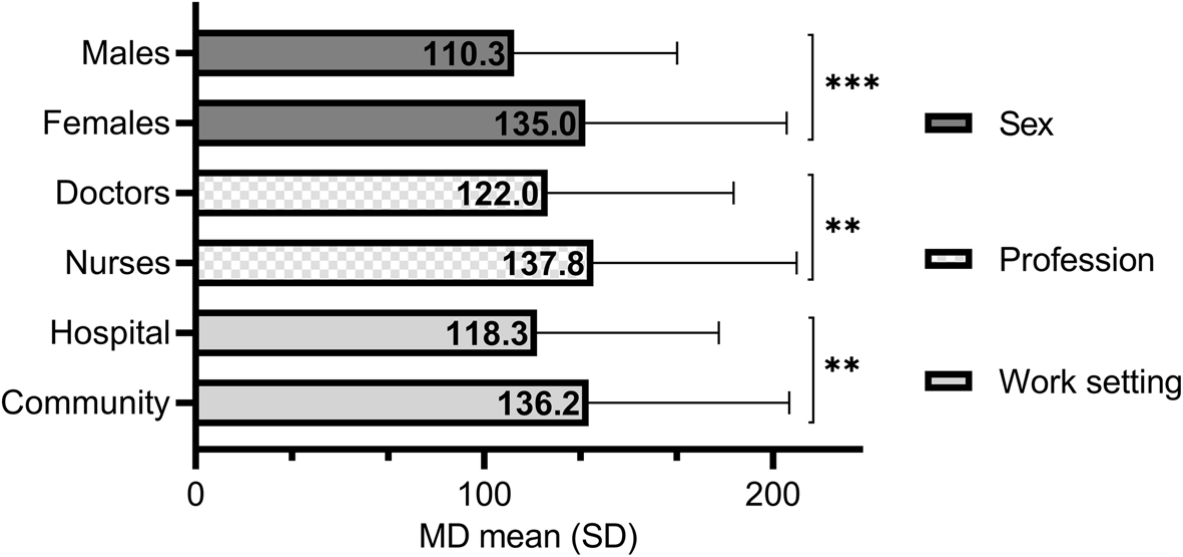
Level of moral distress (MD) according to sex, profession and work setting (**p<0.01; ***p<0.001)

The average value of MD in the total sample was 127.3 ± 66.7 (95% CI 121.8–132.8), with statistically significant differences found based on sex (higher in females), by profession (higher in nurses) and by work setting (higher in the community setting) (Fig. 1).

**Fig. 2:**
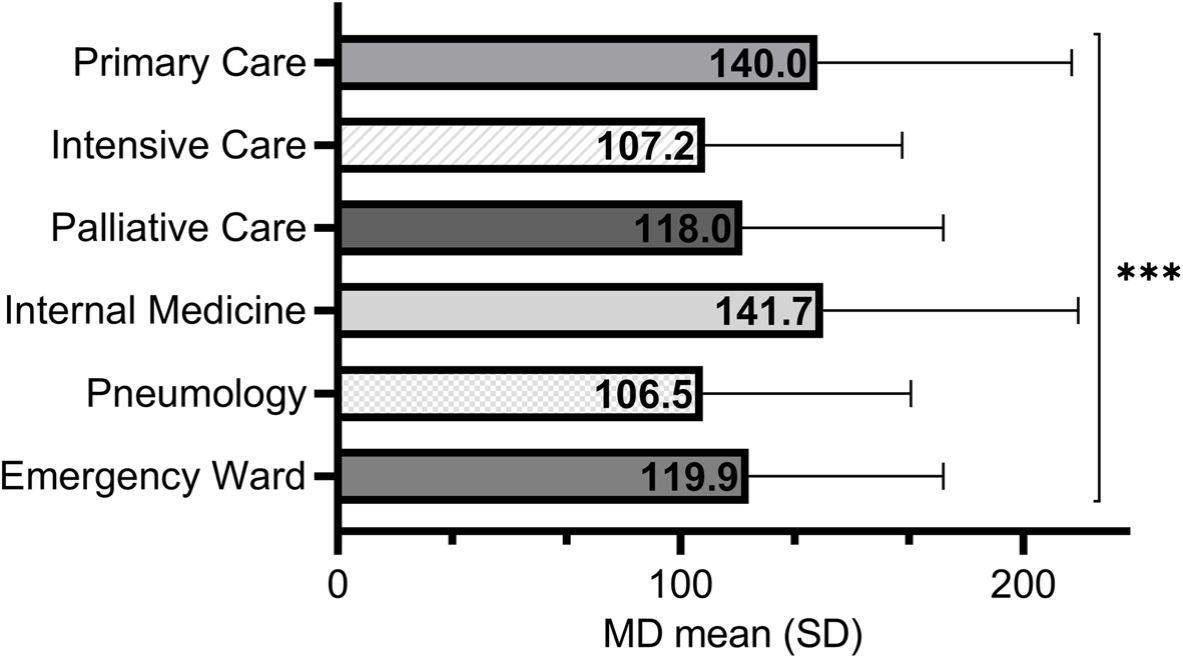
Level of moral distress (MD) according to clinical management unit (***p<0.001)

As regards the comparison between the CMUs (Fig. 2), the post-hoc analysis in the comparison of means showed significant differences between the level of MD among health workers in PHC compared with Pneumology* (p*<0.05) and ICU (*p*<0.001), as well as between Internal Medicine and ICU (*p*<0.05). No significant differences related to age or work experience were found.

### Predictive-associative models of moral distress

Multivariate regressions adjusted for the independent variables which showed statistical significance with MD were carried out, in order to discover the degree of prediction of this phenomenon. Table 2 shows the result of the linear regression model (crude and adjusted) for MD.

**Table 2:** Multivariate analysis: Multiple Linear and Multiple Logistic Regression for moral distress

**Crude Linear Regression (unadjusted)**				
**Variable**	**Beta Coefficient**	**Stand. Beta Coeff.**	**S.E.**	***p***
Age (years)	-0.335	-0.053	0.27	0.209
Work Experience (years)	-0.314	-0.05	0.28	0.261
Sex (Male)	-25.6	-0.173	6.1	<0.001
Profession (Physician)	-14.7	-0.1	6.1	<0.05
Work Setting (Hospital)	-20.5	-0.146	6.1	<0.01
**Adjusted Linear Regression**				
**Variable**	**Beta Coefficient**	**Stand. Beta Coeff.**	**S.E.**	***p***
Constant	154.7		6	
Sex (Male)	-20.2	-0.14	6.1	<0.01
Profession (Physician)	-16.4	-0.115	6.1	<0.01
Work Setting (Hospital)	-20.2	-0.151	5.6	<0.001
Goodness of fit: F = 11.1; *p*<0.001; adjusted *r*^2^ = 0.052				
**Logistic Regression (crude and adjusted)**				
**Variable**	**Orc 95% CI**	***p***	**ORa 95% CI**	***p***
Age (years)	0.995 (0.98 – 1.01)	0.542		
Work Experience (years)	0.99 (0.974 – 1.006)	0.21		
Sex (Female)	2.2 (1.5 – 3.3)	<0.001	2.27 (1.5 -3.4)	<0.001
Profession (Nurse)	1.6 (1.08 – 2.3)	<0.05	1.5 (1.02 -2.2)	<0.05
Work Setting (Community)	1.85 (1.3 – 2.6)	<0.01	2.03 (1.4 -2.9)	<0.01

MMD: 0 (Moral Distress <127.3); 1 (Moral Distress ≧127.3)

Hosmer-Lemeshow: 0.813; r^2^ Nagelkerke: 0.085; r^2^ Cox-Snell: 0.063; Deviance: 721.8

*ORc* Crude Odds Ratio, *ORa* Adjusted Odds Ratio, *SE* Standard Error

As shown in the adjusted linear regression model, the explanatory variable with the highest standardized Beta coefficient was the hospital work setting (-0.151). With all the other variables included in the model being equal, working as a health professional in a hospital setting reduces MD by 20.2 points compared to the community setting, being a male health professional entails a reduction of 20.2 points compared to being a female worker, while working as a physician decreases MD by 16.4 points compared to being a nurse.

As regards predictive capacity, the value of the adjusted determination coefficient (r^2^) was low (0.052), which means the multivariate model should be seen as associative rather than predictive of MD.

Another multivariate model was carried out using multiple binary regression (Table 2). The resultant variable, MD, was dichotomized based on the average value obtained in the sample, in other words, above or below 127.3 points.

The explanatory variable with the highest adjusted OR was sex (female), ORa=2.27 (95% CI 1.5-3.4). With all the other variables included in the model being equal, being a female healthcare professional was 2.27 times more likely to present values of MD above the sample mean (population) than men; working as a nurse had a 1.5 higher risk of obtaining an above-average MD score than physicians; and, finally, being a healthcare professional in the community setting entailed 2.03 times more likelihood of obtaining above-average MD values than in the hospital setting.

The discriminant capacity of the adjusted logistic regression model was measured by calculating the area under the ROC curve, obtaining a value of 0.639 (95% CI 0.59–0.69) *p*<0.001. The model shown by both the discriminant capacity and the coefficients of determination was more associative than predictive.

Based on this logistic regression model adjusted for sex, profession and work setting, the diagnostic accuracy indicators were calculated, obtaining a sensitivity of 45.4%, a specificity of 72.3% and a validity index of 60.6%, while the positive and negative predictive values were 55.6% and 66.4%, respectively.

In addition, the distribution of the level of MD was analysed for each of the dimensions of the MMD-HP SPA scale, according to the explanatory variables significantly associated with MD (Table 3). Of note, the five dimensions produced significantly higher levels of MD in females than in males. Therapeutic obstinacy-futility generated greater MD in health professionals in the hospital environment than in the community setting; however, the external pressure on health workers in the community setting produced greater MD than in the hospital setting. In the healthcare profession, therapeutic obstinacy-futility, interpersonal relationships among the healthcare team and the cover-up of malpractice led to significantly greater MD among nurses than physicians.

**Table 3:** Level of moral distress according to dimensions of the MMD-HP-SPA scale and associated variables.

**Variable**	**Global** Mean (SD)	**Males** Mean (SD)	**Females** Mean (SD)	***p***	**Nurses** Mean (SD)	**Physicians** Mean (SD)	***p***	**Hospital** Mean (SD)	**Community** Mean (SD)	***p***
Health Care	47.7 (25.5)	43.7 (23)	49.2 (25.9)	<0.05	46 (24.2)	48.3 (25.5)	0.3	40.5 (22.6)	53.7 (26.3)	<0.001
Therapeutic Obstinacy-Futility	22.9 (15)	18.8 (12.9)	24.4 (15.4)	<0.001	28.6 (16.5)	19.6 (12.9)	<0.001	24.5 (15.6)	21.5 (14.4)	<0.05
Interpersonal Relations with Healthcare Team	18.6 (14.7)	15.9 (13)	20.2 (15)	<0.01	21.9 (17.3)	17.3 (14.2)	<0.001	17.6 (14.1)	19.6 (14.9)	0.172
External Pressure	20.3 (15.3)	15.8 (12.5)	22.2 (15.8)	<0.001	21.3 (15.4)	19.6 (14.9)	0.219	17 (13.7)	23 (16)	<0.001
Covering up Medical Malpractice	11.3 (9.5)	9.6 (8.2)	12.3 (9.9)	<0.01	12.8 (10)	10.8 (9.1)	<0.05	10.9 (9.7)	11.7 (9.4)	0.321

### Main sources of moral distress

Finally, the main causes of MD among the health professionals were analysed. Table 4A and B show the ten most relevant sources together with the highest mean values segregated by the variables significantly associated with MD and classified (see ‘rank’). The maximum value for any cause (item) was 16 points.

**Table 4:** Main sources of moral distress in health professionals

A									
**Source of Moral Distress**	**Dimension**	**Global** **Mean (SD)**	**Rank**	**Males** **Mean (SD)**	**Rank**	**Females** **Mean (SD)**			**Rank**
Source of Moral Distress	Dimension	Global Mean (SD)	Rank	Males Mean (SD)	Rank	Females Mean (SD)			Rank
16^a^. Having to attend to more patients than I can safely attend to.	Health Care	9.8 (4.9)	1	9.1 (5)	1	10.1 (4.8)			1
17. Seeing how patient care is negatively affected by the lack of resources/equipment or the availability of beds.	Health Care	8.3 (4.7)	2	7.9 (4.5)	2	8.5 (4.8)			2
9. Seeing how patient care suffers due to the lack of continuity of care.	Health Care	7.4 (4.5)	3	6.6 (4.2)	4	7.7 (4.7)			3
19. Having to deal with excessive paperwork, which negatively affects patient care.	Health Care	7.1 (4.9)	4	6.9 (4.8)	3	7.2 (5)			5
18. Witnessing inaction or lack of administrative support on an issue that negatively affects patient care.	Health Care	7 (4.7)	5	6.5 (4.2)	5	7.3 (4.8)			4
22. Having to work with aggressive or rude patients or family members who negatively affect the quality of care.	External Pressure	6 (4.6)	6	5.1 (4.1)	7	6.4 (4.7)			6
23. Feeling compelled to place too much emphasis on skills and productivity or quality measures at the expense of patient care.	Health Care	5.97 (4.8)	7	5.3 (4.6)	6	6.2 (4.9)			7
3. Feeling pressure to give/carry out orders which I consider unnecessary or to administer inappropriate tests or treatments.	Therapeutic Obstinacy-futility	5.7 (4.3)	8	4.6 (3.9)	8	6.1 (4.4)			8
2. Accepting the family’s insistence to continue an aggressive treatment, even though I believe it is not the best treatment for the patient.	Therapeutic Obstinacy -futility	5.1 (4)	9	4.4 (3.8)	9	5.4 (4.1)			9
4. Being unable to provide optimal care due to pressure from administrators or insurers to reduce costs.	Health Care	5 (4.9)	10	4.4 (4.6)	10	5.3 (5.1)			10
B									
**Source of Moral Distress**	**Dimension**	**Nurses** **Mean (SD)**	**Rank**	**Physicians** **Mean (SD)**	**Rank**	**Hospital** **Mean (SD)**	**Rank**	**Community** **Mean (SD)**	**Rank**
16^a^. Having to attend to more patients than I can safely attend to.	Health Care	9.5 (4.7)	1	9.9 (5)	1	8.6 (4.7)	1	10.9 (4.8)	1
17. Seeing how patient care is negatively affected by the lack of resources/equipment or the availability of beds.	Health Care	7.9 (4.6)	2	8.5 (4.8)	2	7.4 (4.6)	2	9 (4.8)	2
9. Seeing how patient care suffers due to the lack of continuity of care.	Health Care	7.2 (4.6)	3	7.4 (4.5)	3	6.5 (4.2)	3	8.1 (4.79	4
19. Having to deal with excessive paperwork, which negatively affects patient care.	Health Care	6.4 (4.5)	6	7.4 (4.8)	5	5.9 (4.7)	5	8.1 (5)	3
18. Witnessing inaction or lack of administrative support on an issue that negatively affects patient care.	Health Care	6.5 (4.5)	5	7.3 (4.8)	4	6.2 (4.6)	4	7.6 (4.8)	5
22. Having to work with aggressive or rude patients or family members who negatively affect the quality of care.	External Pressure	6.4 (4.6)	8	5.8 (4.5)	6	4.9 (4.2)	10	7.1 (4.8)	6
23. Feeling compelled to place too much emphasis on skills and productivity or quality measures at the expense of patient care.	Health Care	6.4 (4.8)	9	5.8 (4.8)	7	5.2 (4.5)	9	6.8 (5.1)	7
3. Feeling pressure to give/carry out orders which I consider unnecessary or to administer inappropriate tests or treatments.	Therapeutic Obstinacy-futility	6.7 (4.3)	4	5.8 (4.5)	8	5.4 (4.3)	7	6 (4.4)	9
2. Accepting the family’s insistence to continue an aggressive treatment, even though I believe it is not the best treatment for the patient.	Therapeutic Obstinacy-futility	6.4 (4.4)	10	4.5 (3.6)	10	5.7 (4.1)	6	4.8 (4.4)	11
4. Being unable to provide optimal care due to pressure from administrators or insurers to reduce costs.	Health Care	4.8 (4.7)	11	5.1 (5)	9	3.5 (4.4)	11	6.3 (5.2)	8

^a^Numbers refer to items. Rank: Ranking (order)

70% of the sources of MD belonged to the dimension of "Health Care", and the top five causes with the highest average value belonged to this dimension. The cause *"Having to care for more patients than I can safely care for"* (item no. 16) obtained the highest average value (Mean=9.8; SD=4.9) in the whole sample and was also the cause of the greatest MD in each of the three associated explanatory variables. It was also the highest average source of MD among health professionals in the community (Mean=10.9; SD=4.8) and in women (Mean=10.1; SD=4.8).

## Discussion

The MD experienced by health professionals is an integral part of their profession. It was heightened during the pandemic, due to the tension that arose between the standard pre-pandemic clinical ethics used by professionals and the public health ethics imposed after the onset of the pandemic. When the pandemic broke out, this approach suddenly but inevitably changed, despite the innate predisposition among professionals to maintain respect for patient autonomy in decision-making, minimise harm and maximise benefit, and focus on the most vulnerable patients [27]. Some of these principles came into conflict during the emergency global health crisis of the COVID-19 pandemic [28].

In the present work, high levels of average MD were found (127.3). While it is true that the MMD-HP scale is relatively recent and there are no cut-off points that allow the MD of health professionals to be classified as high or low [19], however, comparing these results with other studies carried out with the same scale, higher mean scores were found in the present sample than in most (108.9 in physicians, nurses and other health personnel [19]; 93.4 in pre-pandemic paediatricians in the USA [7]; 98.2 in physicians and nurses in Japan [20]; 122.8 in ICU nurses in Japan [29]; 107 in ICU physicians in Canada [30]; 116.52 in ICU nurses in Greece [31]; 117.57 in Canadian health professionals [17]; and a mean of 68 in ICU physicians and nurses in Spain [21]. Nevertheless, there are some published studies with slightly higher levels of MD using the MMD-HP [32–34]).

Clearly, the emotional burnout suffered in the COVID-19 pandemic seems to have taken its toll on healthcare professionals around the world, and there is evidence that levels of MD have risen among these professionals [2, 16, 17]. In addition to the shortcomings in health care, another decisive factor was not being able to give the necessary emotional support to suffering patients, due to the strict preventive measures or even having to self-isolate after coming into close contact with the virus, when colleagues were in urgent need of help. Our results may partly reflect this distress, since the surveys were carried out almost 2 years into the pandemic, and its effects were still being felt. Unfortunately, it was no possible to compare the findings of the current study with pre-pandemic results, since no data from earlier measurements with this scale were available, but it is widely accepted that the level of distress has increased as a result of the appearance of SARS-CoV-2 Coronavirus and specific causes of moral distress have become more prevalent or distressing [2, 16, 17, 21]. It is also clear that health professionals who worked with COVID patients showed significantly higher levels of MD than those who did not [25], although there is one surprising study in which lower levels of moral distress in nurses and intensive care providers compared to a control group one year before COVID-19 were reported [23].

The first of the consolidated findings in this study is the higher level of MD in females compared to males, although few authors have analysed this variable independently. Spilg et al. [25] showed that being male was one of the factors independently associated with greater moral resilience and Malliarou et al. [31] found that females had higher scores on the MMD-HP scale. Indirectly, many other studies are in line with this conclusion, since almost all the published works show that MD is higher in nurses than in physicians [23, 32, 34, 35] and it is an undisputable fact that there is a higher proportion of females in the nursing profession than in the medical profession. Although it is beyond the scope of this study to hypothesize why MD is greater in females, these results could be justified by a meta-analysis of 19 studies on sensitivity and moral judgment which showed significantly higher scores among women, and according to these authors, the differences in the development of moral sensitivity have been consistently reported in the developmental psychology literature [36]. Professional experience could be a factor to be considered in this regard, although there is contradictory evidence. Some studies show a positive relationship between years of experience and MD levels in relation to the so-called “crescendo effect” [37, 38], while others report the opposite [26]. However, in line with previous studies [39–41], the present work has not found that years of experience are associated with MD levels. What is relevant to this study is the higher risk that may arise in the future due to the increasing proportion of females among health professionals, so that if being female is the greatest predictor of MD (OR=2.27), urgent strategies need to be devised which include the gender perspective [42] and attempt to reduce this distress.

Ruston et al. [43] have written extensively on MD coping strategies, including religious beliefs or feelings of spirituality, as well as level of moral resilience, the latter of which moderate the inverse relationship between the number of years of professional experience and moral injury. Morley et al. [44], in a systematic review identified the following interventions to mitigate MD: educational interventions, facilitated discussions ranging from 30 to 60 minutes, specialist consultation services, multidisciplinary rounds, self-reflection and narrative writing.

As regards the profession, nearly all the studies with the MMD-HP scale coincide that MD is higher in nurses than in physicians [23, 32, 34, 35], with the exception of Rodriguez-Ruiz et al. [21, 24, 26]. The results of the present study show mean MD values of 137.8 for nurses compared to 122 for physicians, which is in line with most published studies. The fact that nurses tend to have a lower level of autonomy than physicians seems to be a logical explanation for this phenomenon, and this is a factor closely related to anxiety and the level of MD. Nursing staff have to implement therapeutic measures prescribed by physicians, even though they may not agree with their application, and so they have less decision-making capacity in this regard. According to Corley et al. [45] nurses tend to have more responsibility than authority, since they have less power in the health institutions where they work, which is why they are often expected to follow orders although they may disagree with them on moral grounds [46].

The work setting and clinical unit of origin have not been analysed in many publications. Beck et al. reported a mean score of 93.4 in paediatricians [7], while Fujii et al. provided data from other healthcare professionals from settings other than ICUs, finding lower MD levels in medical wards, followed by surgical wards, with higher levels in ICUs/emergency wards [20]. Similar findings were reported by Bayanzay et al. [34]. In the present study, there were significant differences between the different CMUs and work (community/hospital) settings analysed, with higher levels in Internal Medicine (141.7) and in PHC (140), but lower levels in ICUs (107.2) and Pneumology (106.5), as well as clearly higher levels in the community setting (137.5) than in hospitals (117.1). Unfortunately, the findings of the current study could not be compared with other research due to the lack of comparable studies mentioned above, although some of our results may be related to the specific time period (starting in the pandemic). In fact, the main cause of MD for all the categories analysed was Item 16 (“*Having to attend to more patients than I can safely attend to*”), which was far ahead of Item 17 (“*Seeing how patient care is negatively affected by the lack of resources/equipment or the availability of beds*”). This can certainly explain why certain specialities, specifically in the community setting, which is made up mostly of PHC health workers, are overwhelmed by issues as obvious as the high pressure of care (which is greater in PHC than in the hospital setting) or the growing waiting lists. Patterson et al. recognized the group of PHC professionals as particularly affected because they were considered "essential" compared to other colleagues, regardless of the setting and clinical circumstances [47]. According to our results, higher scores in MD in the community setting are explained mainly by sources becoming of the dimension “health care”, particularly items 16 (to care more patients than they can), 9 (lack of continuity of care), and 4 (unable to provide optimal care due to pressure of administrators to reduce costs). Very few studies have compared different clinical settings including primary care, particularly among different healthcare professionals. Nevertheless, Giannetta et al. [48] have made a scoping review in nurses and identify the factors that trigger moral distress, many of which are related to everyday life, such as poor organization of the working process; conflicting interpersonal relationships among the patient, the community, and the healthcare professionals; and particularly during the providing end-of-life care at the patient’s own home.

On the other hand, at this point after the pandemic, there is no longer a severe shortage of resources such as respiratory support equipment, ICU beds or personal protective equipment, nor is there such a great need to postpone care for other health problems, as described by Rubio et al. [15].

Regarding the main sources of MD, 70% of the sources of MD were in the Health Care dimension, with the top five causes with the highest average value belonging to this dimension. The cause "*Having to attend to more patients than I can safely attend to*" (Item 16), obtained the highest average value (Mean=9.8; SD=4.9) for the entire sample, and was also the greatest cause of MD in each of the three associated explanatory variables. The results of the current study regarding the main sources of MD are almost identical to the findings of Rodriguez-Ruiz et al. [24]. The first 3 causes coincide in the same order (Items 16, 17 & 9), while the 4th cause (Item 19 in the survey) was ranked 5th by Rodriguez-Ruiz et al. [24]. All these causes fall under the dimension of ‘Health Care’, which in their case was defined as ‘causes related to patients’. The findings of the present work also partially coincide with Bayanzay et al. [34] for nurses (for whom Item 16 had the highest value), although not for physicians, who rated Item 2 as the most stressful (“*Accepting the family’s insistence to continue an aggressive treatment, even though I believe it is not the best treatment for the patient*”), which in this case was ranked as the tenth cause of MD. In contrast, Bleicher et al. [32] and Ashida et al. [29] found that the items that caused greater MD were those related to the factor of ‘Therapeutic obstinacy/futility’. In the current study, ‘Therapeutic obstinacy/futility’ generated greater MD in health professionals in the hospital than in the community setting; however, external pressure on health workers in the community setting produced greater MD than in the hospital setting. With respect to the healthcare profession, therapeutic obstinacy/futility, interpersonal relations with the healthcare team and the cover-up of malpractice caused significantly greater MD in nurses compared to physicians.

A limitation of the study is the low representativeness of the sample due to consecutive sampling instead of stratified random sampling, which would have allowed a better homogeneity of the groups.

## Conclusions

Being female, being a nursing professional, and working in the community setting presented a higher risk of moral distress. Being a female health professional was 2.27 times more likely to present values of moral distress above the mean of the sample than being a male; working as a nurse resulted in a 1.5 greater risk of obtaining an above-average moral distress score than working as a physician and working in the community setting entailed 2.03 times more risk of moral distress than in the hospital.

70% of the sources of moral distress were in the Health Care dimension, with the top five causes with the highest average value belonging to this dimension. The cause *"Having to attend to more patients than I can safely attend to"* (Item 16), obtained the highest average value, and was also the greatest cause of moral distress in each of the three associated explanatory variables.

To alleviate moral distress among healthcare professionals, the current health model needs to implement an ethical approach to public health issues and implement measures that take into account the gender perspective, the reconciliation of family life and care overload, among others.

## Data Availability

The datasets used and/or analysed during the current study are available from the corresponding author on reasonable request.

## Abbreviations:

ANOVA: Analysis of Variance
CMU: Clinical Management Unit
ICU: Intensive Care
IQR: Interquartile Range
MD: Moral Distress
MMD-HP: Measure of Moral Distress for Healthcare Professionals
MMD-HP-SPA: Spanish version of the Measure of Moral Distress for Health Care Professionals
MDS-R: Moral Distress Scale-Revised
ORc: Crude Odds Ratio
ORa: Adjusted Odds Ratio
PHC: Primary Health Care
ROC curve: Receiver Operating Characteristic curve
SE: Standard Error
